# Peripheral doses in patients undergoing Cyberknife treatment for intracranial lesions. A single centre experience

**DOI:** 10.1186/1748-717X-6-157

**Published:** 2011-11-14

**Authors:** Vassiliki Vlachopoulou, Christos Antypas, Harry Delis, Argyrios Tzouras, Nikolaos Salvaras, Dimitrios Kardamakis, George Panayiotakis

**Affiliations:** 1 Department of Medical Physics, School of Medicine, University of Patras, Achaia, Greece; 2 CyberKnife Center, Iatropolis, Athens, Attica, Greece; 3 Department of Radiology, School of Medicine, University Hospital of Patras, Achaia, Greece

**Keywords:** Cyberknife, intracranial lesions, MOSFET, peripheral dose, stereotactic/radiosurgery radiotherapy

## Abstract

**Background:**

Stereotactic radiosurgery/radiotherapy procedures are known to deliver a very high dose per fraction, and thus, the corresponding peripheral dose could be a limiting factor for the long term surviving patients. The aim of this clinical study was to measure the peripheral dose delivered to patients undergoing intracranial Cyberknife treatment, using the MOSFET dosimeters. The influence of the supplemental shielding, the number of monitor units and the collimator size to the peripheral dose were investigated.

**Methods:**

MOSFET dosimeters were placed in preselected anatomical regions of the patient undergoing Cyberknife treatment, namely the thyroid gland, the nipple, the umbilicus and the pubic symphysis.

**Results:**

The mean peripheral doses before the supplemental shielding was added to the Cyberknife unit were 51.79 cGy, 13.31 cGy and 10.07 cGy while after the shielding upgrade they were 38.40 cGy, 10.94 cGy, and 8.69 cGy, in the thyroid gland, the umbilicus and the pubic symphysis, respectively. The increase of the collimator size corresponds to an increase of the PD and becomes less significant at larger distances, indicating that at these distances the PD is predominate due to the head leakage and collimator scatter.

**Conclusion:**

Weighting the effect of the number of monitor units and the collimator size can be effectively used during the optimization procedure in order to choose the most suitable treatment plan that will deliver the maximum dose to the tumor, while being compatible with the dose constraints for the surrounding organs at risk. Attention is required in defining the thyroid gland as a structure of avoidance in the treatment plan especially in patients with benign diseases.

## Background

During all radiotherapy treatments, there is always a small unavoidable fraction of the delivered dose that is absorbed by radiosensitive tissues/organs outside the irradiated volume, known as peripheral dose (PD). PD is due to radiation that is scattered from the patients' body, the linac head components, the treatment room walls and lastly, radiation leakage from the linac head. Stereotactic radiosurgery/radiotherapy (SRS/SRT) procedures main aim is to deliver a very high dose per fraction to the target, and thus the corresponding PD outside the treatment volume is an important issue, especially for the long term surviving patients.

The main concern in a treatment plan is on how to apply the maximum dose to the target, without exceeding the dose constraints of the surrounding organs at risk. These dose constraints, which are based on clinical studies, aim at minimizing side effects (normal tissue complications), which could even be the induction of secondary cancer [1]. The risk for secondary cancer is of a main concern especially in long term surviving patients, who are treated for benign diseases or for curatively non metastatic malignancies. Epidemiological evidence from human populations demonstrate that organ doses above 5-10 cGy for protracted exposures, or 1-5 cGy, for acute exposures, could increase the risk of some types of cancer [2].

The thyroid gland is a very radiosensitive organ that, although is not the target during intracranial treatments, it can be affected by scattered radiation [3]. Especially, in young patients, it has been shown that there is a significantly increased risk of cancer in the thyroid gland, after exposure to radiation, as part of therapy in childhood cancers [4]. The breast is also one of the sensitive organs regarding the carcinogenic effects of radiation, and there is an excessive risk of secondary cancers being induced for the breast even at doses as low as 1-9 cGy [2].

The Cyberknife is a frameless, image-guided, stereotactic radiosurgery system with sub-millimeter clinical accuracy [5]. The system comprises of a 6 MV linear accelerator mounted on a robotic arm, along with an image guided system. Through the image guidance cameras (which are composed of a pair of orthogonal diagnostic x-ray tubes and corresponding image detectors), specialized software, which uses x-ray images obtained throughout the treatment, verifies the patient position, based on radiographic landmarks, such as fiducials [6], skull anatomy [7] or spine anatomy landmarks [8]. After the initial setup of the patient, when the tumor is localized and aligned, the radiation is delivered. The treatment is modified in real time to compensate for tumor movements. Several hundred treatment beams are chosen out of a repertoire of more than one thousand possible beam directions, using inverse treatment planning. These beams are delivered in a non-isocentric manner via small circular fields of varying size and weighted with different monitor units (MU) [9].

To our knowledge, there have not been any other reports of PD in patients undergoing intracranial treatment with Cyberknife using Metal Oxide Semiconductor Field Effect Transistor (MOSFET) dosimeters in the literature. However, there have been previous reports that have studied PD in stereotactic radiosurgery and radiotherapy treatments [10-14].

MobileMOSFET seems to be an appropriate dosimetry system for in vivo measurements of low peripheral doses during stereotactic radiosurgery/radiotherapy due to its extremely small size (active area 0.04 mm^2^), and its simple and immediate read out, compared to the thermoluminescent dosimeter, and its accuracy at low doses [15-21].

The aim of this study was to evaluate PD at preselected anatomical areas on the patient skin, corresponding to radiosensitive organs using the MOSFET dosimeters and to investigate the influence of the supplemental shielding, the number of MU and the collimator size on the PD.

## Methods

### Patient information

In a study of 31 patients (fourteen (14) men and seventeen (17) women), that underwent intracranial Cyberknife treatment at the Iatropolis Cyberknife Center of Athens, PD was measured on four preselected areas of the body, namely, the thyroid gland, the nipple, the umbilicus and the pubic symphysis, using the mobileMOSFET dose verification system [22]. These measurements took place in two different chronological periods, before and after the shielding upgrade of the Cyberknife unit; consequently ten patients were treated before the installation of supplemental shielding and the remainder after the upgrade. The MOSFET dosimeters were calibrated before their use under reference conditions. The histological diagnoses of the patients are shown in Table 1.

**Table 1:** Histologies of patients undergoing Cyberknife treatment for intracranial or cranial lesions.

HISTOLOGIES	NUMBER OF CASES
Acoustic neuromas	7
Arterial venous malformations	2
Bone metastases	3
Brain metastases	6
Craniopharyngioma	1
Gliomas	3
Meningiomas	6
Nasopharyngeal carcinoma	1
Pituitary adenoma	1
T-lymphoma	1

### Cyberknife treatment

**Figure 1:**
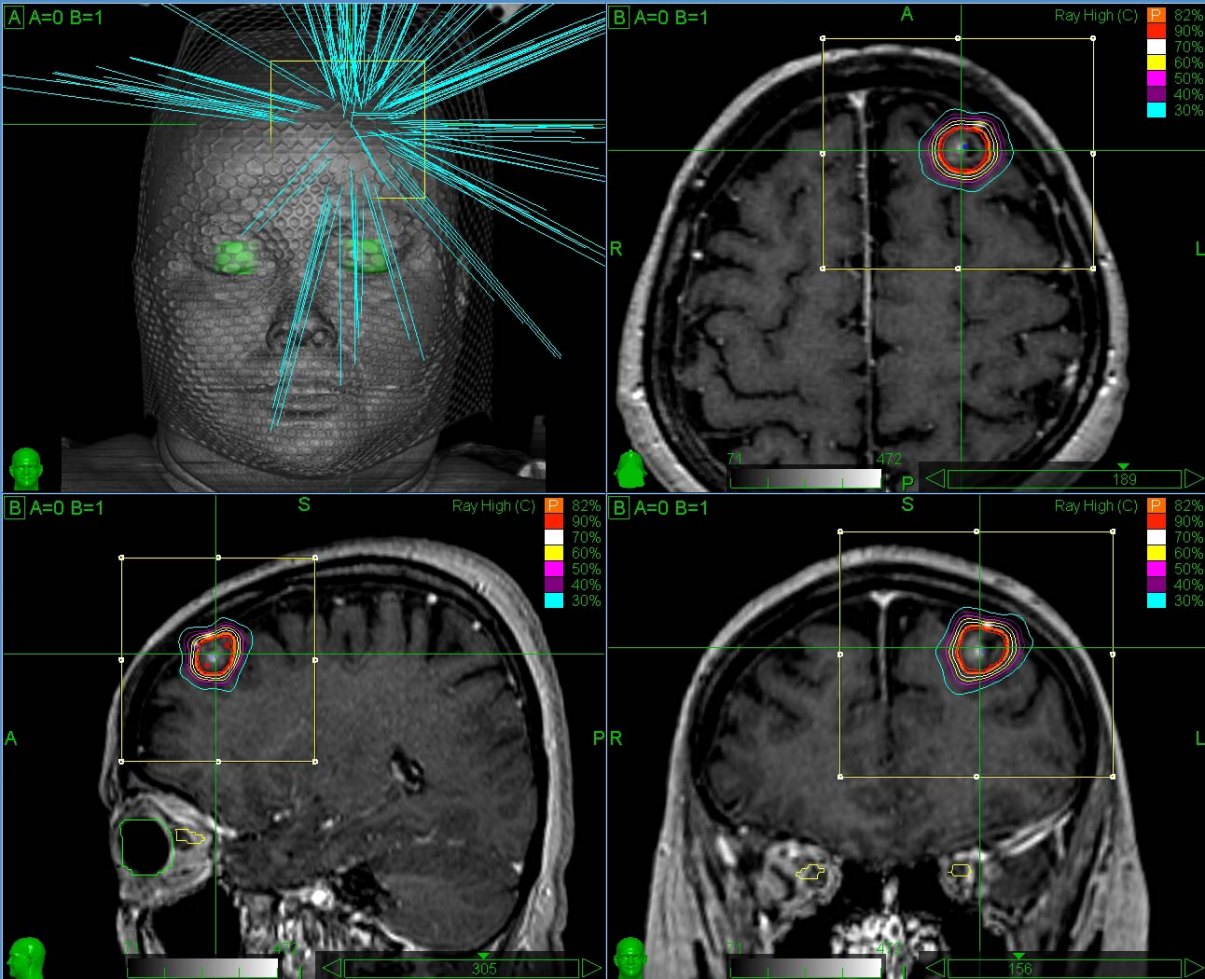
**Illustration of the Cyberknife treatment beam geometry and of the treatment plan in axial, coronal, and sagittal views**.

Tumor and organs at risk were visualized and delineated on a volumetric CT study of each patient. During the treatment planning process (Multiplan treatment planning system v.1.7), a finite set of non-isocentric, non-coplanar treatment beams were created, producing the final dose distribution (Figure 1). Each treatment beam is correlated to a source point in a space around the patient, through which the photon beam is directed towards the target volume. Each source point is called a node and the complete set of nodes constitutes the treatment path [23]. For each patient one to three different path sets (namely "even path head", "1 path head" and "short path head") were utilized.

Different treatment field sizes, determined by interchangeable secondary circular collimators with diameters of 5.0, 7.5, 10.0, 12.5, 15.0, 20.0, 25.0, 30.0, 35.0 and 60.0 mm (defined at a nominal treatment distance of 80 cm) can be assigned to each treatment path. The prescribed dose (TD) that is delivered to each patient is from one to five fractions, using the Cyberknife system of version 7.5, with a 600 MU/min linac.

Additional 19 mm (0.75 inch) tungsten shielding, was installed by the manufacturer, at the point where the primary collimator housing narrows to hold the secondary collimator as described by Chuang et al [12]. The purpose of the shielding was to reduce the leakage radiation, leading to a decrease of PD.

For all treatments studied, the 6D Skull tracking method is utilized, which can be used for both intracranial targets and head and neck targets that can be considered fixed relatively to the skull. Image registration is performed using high contrast bone information contained within the entire field of view. The resulting 2D transformations for each orthogonal projection are combined and backprojected to determine the 3D rigid transformation that aligns the position and orientation of the skull in the treatment planning CT image with the treatment delivery coordinate system [7,23].

## Results

### Peripheral dose measurements

Results of the PD measurements in the four extracranial sites, before and after shielding, are presented in Tables 2, 3, 4, 5, 6, 7 and 8. The distance between the point of measurement (the active area of MOSFET dosimeters) and the treatment center was calculated with respect to the room lasers. The distance ranged from 14 to 22 cm for the thyroid gland, 29 to 42 cm for the nipple, 52 to 64 cm for the umbilicus and 63 to 88 cm for the pubic symphysis.

**Table 2:** Preshielding peripheral dose in the region of the thyroid gland, expressed as a percentage of the monitor units and as a percentage of the prescribed dose.

Collimator size (mm)	Distance (cm)	Preshielding peripheral dose (cGy)	Preshielding peripheral dose (%MU)	Preshielding peripheral dose (%TD)	Monitor units	Prescribed dose (cGy)	Isodose line (%)
12.5	20	5.00	0.12	0.25	4005	2000	84
10	20	6.48	0.12	0.32	5587	2000	75
20, 20, 20	22	15.22	0.13	0.76	11374	2000	75
5, 5, 5	20	23.01	0.13	1.15	18026	2000	75
7.5, 7.5, 15	16.5	35.38	0.17	1.97	20487	1800	79
10, 10, 15	14	164.90	0.69	6.60	23876	2500	75
20, 20, 20	14	40.55	0.16	1.62	25794	2500	67
5, 5, 5	18	59.43	0.22	3.30	27433	1800	76
7.5, 7.5, 15	19	35.73	0.13	1.99	28411	1800	75
7.5, 15, 25	14.5	132.15	0.33	5.29	39476	2500	70

MU: Monitor Units

TD: Prescribed Dose

**Table 3:** Postshielding peripheral dose in the region of the thyroid gland, expressed as a percentage of the monitor units and as a percentage of the prescribed dose.

Collimator size (mm)	Distance (cm)	Postshielding peripheral dose (cGy)	Postshielding peripheral dose (%MU)	Postshielding peripheral dose (%TD)	Monitor units	Prescribed dose (cGy)	Isodose line (%)
12.5	22	3.11	0.09	0.14	3483	2200	80
25	16	32.80	0.32	1.64	10355	2000	70
20, 20, 10	17	19.80	0.15	1.24	12911	1600	70
12.5, 12.5, 10	16	38.36	0.29	2.95	13271	1300	70
20, 20, 7.5	15	27.50	0.19	1.96	14289	1400	70
15	15	29.50	0.20	1.97	14621	1500	70
35	20	20.37	0.14	1.13	15061	1800	70
12.5, 12.5, 7.5	20	17.60	0.09	0.98	20391	1800	70
10	20	12.13	0.06	0.67	20789	1800	70
5, 5, 5	18	59.44	0.29	2.97	20798	2000	70
7.5	18	14.19	0.07	0.65	20969	2200	70
10, 10, 5	17	50.40	0.21	2.40	24249	2100	70
7.5, 15, 15	16.5	59.64	0.23	3.31	25696	1800	70
10, 12.5, 20	19	19.79	0.07	1.24	26656	1600	75
5, 5, 10	19	23.13	0.08	1.29	29230	1800	70
5, 10, 12.5	19	45.80	0.15	2.41	29833	1900	70
5, 5, 5	17	55.80	0.17	2.66	32202	2100	70
7.5, 12.5, 20	16	160.88	0.49	8.04	33130	2000	70
5, 7.5, 12.5	18	35.22	0.08	1.96	42177	1800	70
7.5, 12.5, 12.5	18	42.45	0.10	1.57	43623	2700	75

MU: Monitor Units

TD: Prescribed Dose

**Table 4:** Postshielding peripheral dose in the region of the nipple, expressed as a percentage of the monitor units and as a percentage of the prescribed dose.

Collimator size (mm)	Distance (cm)	Postshielding peripheral dose (cGy)	Postshielding peripheral dose (%MU)	Postshielding peripheral dose (%TD)	Monitor units	Prescribed dose (cGy)	Isodose line (%)
12.5	38	1.34	0.04	0.06	3483	2200	80
25	29	6.04	0.06	0.30	10355	2000	70
20, 20, 10	33	7.89	0.06	0.49	12911	1600	70
12.5, 12.5, 10	31	7.22	0.05	0.56	13271	1300	70
20, 20, 7.5	31	7.96	0.06	0.57	14289	1400	70
15	31	7.75	0.05	0.52	14621	1500	70
35	37	8.49	0.06	0.47	15061	1800	70
12.5, 12.5, 7.5	36	11.84	0.06	0.66	20391	1800	70
10	38	11.45	0.06	0.64	20789	1800	70
5, 5, 5	32	11.24	0.05	0.56	20798	2000	70
7.5	35	10.54	0.05	0.48	20969	2200	70
10, 10, 5	29.5	11.67	0.05	0.56	24249	2100	70
5	31	13.65	0.06	0.76	24680	1800	80
7.5, 15, 15	32	14.79	0.06	0.82	25696	1800	70
10, 12.5, 20	33	13.24	0.05	0.83	26656	1600	75
5, 5, 10	34	12.24	0.04	0.68	29230	1800	70
5, 10, 12.5	35	13.20	0.04	0.69	29833	1900	70
5, 5, 5	33	16.02	0.05	0.76	32202	2100	70
7.5, 12.5, 20	32	20.12	0.06	1.01	33130	2000	70
5, 7.5, 12.5	34	23.10	0.05	1.28	42177	1800	70
7.5, 12.5, 12.5	42	26.07	0.06	0.97	43623	2700	75

MU: Monitor Units

TD: Prescribed Dose

**Table 5:** Preshielding peripheral dose in the region of the umbilicus, expressed as a percentage of the monitor units and as a percentage of the prescribed dose.

Collimator size (mm)	Distance (cm)	Preshielding peripheral dose (cGy)	Preshielding peripheral dose (%MU)	Preshielding peripheral dose (%TD)	Monitor units	Prescribed dose (cGy)	Isodose line (%)
12.5	58	2.15	0.05	0.11	4005	2000	84
10	58	2.83	0.05	0.14	5587	2000	75
20, 20, 20	62	7.67	0.07	0.38	11374	2000	75
5, 5, 5	59	11.64	0.06	0.58	18026	2000	75
7.5, 7.5, 15	59	12.62	0.06	0.70	20487	1800	79
10, 10, 15	58	16.00	0.07	0.64	23876	2500	75
20, 20, 20	55	19.35	0.08	0.77	25794	2500	67
5, 5, 5	56	17.40	0.06	0.97	27433	1800	76
7.5, 7.5, 15	55	16.68	0.06	0.93	28411	1800	75
7.5, 15, 25	50	26.75	0.07	1.07	39476	2500	70

MU: Monitor Units

TD: Prescribed Dose

**Table 6:** Postshielding peripheral dose in the region of the umbilicus, expressed as a percentage of the monitor units and as a percentage of the prescribed dose.

Collimator size (mm)	Distance (cm)	Postshielding peripheral dose (cGy)	Postshielding peripheral dose (%MU)	Postshielding peripheral dose (%TD)	Monitor units	Prescribed dose (cGy)	Isodose line (%)
12.5	63	1.24	0.04	0.06	3483	2200	80
25	55	6.20	0.06	0.31	10355	2000	70
20, 20, 10	57	6.44	0.05	0.40	12911	1600	70
12.5, 12.5, 10	55	6.89	0.05	0.53	13271	1300	70
20, 20, 7.5	54	7.36	0.05	0.53	14289	1400	70
15	52	6.75	0.05	0.45	14621	1500	70
35	65	8.22	0.05	0.46	15061	1800	70
12.5, 12.5, 7.5	62.5	8.86	0.04	0.49	20391	1800	70
10	67	8.59	0.04	0.48	20789	1800	70
5, 5, 5	55	8.96	0.04	0.45	20798	2000	70
7.5	62	9.65	0.05	0.44	20969	2200	70
10, 10, 5	58.5	14.25	0.06	0.68	24249	2100	70
5	60	10.05	0.04	0.56	24680	1800	80
7.5, 15, 15	54	12.57	0.05	0.70	25696	1800	70
10, 12.5, 20	58	13.48	0.05	0.84	26656	1600	75
5, 5, 10	56	14.22	0.05	0.79	29230	1800	70
5, 10, 12.5	58	13.54	0.05	0.71	29833	1900	70
5, 5, 5	63	15.24	0.05	0.73	32202	2100	70
7.5, 12.5, 20	57	17.52	0.05	0.88	33130	2000	70
5, 7.5, 12.5	62	20.07	0.05	1.12	42177	1800	70
7.5, 12.5, 12.5	64	19.68	0.05	0.73	43623	2700	75

MU: Monitor Units

TD: Prescribed Dose

**Table 7:** Preshielding peripheral dose in the region of the pubic symphysis, expressed as a percentage of the monitor units and as a percentage of the prescribed dose.

Collimator size (mm)	Distance (cm)	Preshielding peripheral dose (cGy)	Preshielding peripheral dose (%MU)	Preshielding peripheral dose (%TD)	Monitor units	Prescribed dose (cGy)	Isodose line (%)
12.5	82	1.42	0.04	0.07	4005	2000	84
10	82	2.36	0.04	0.12	5587	2000	75
20, 20, 20	77	5.54	0.05	0.28	11374	2000	75
5, 5, 5	73	11.10	0.06	0.56	18026	2000	75
7.5, 7.5, 15	80	8.69	0.04	0.48	20487	1800	79
10, 10, 15	71	20.15	0.08	0.81	23876	2500	75
20, 20, 20	75	11.45	0.04	0.46	25794	2500	67
5, 5, 5	70	14.70	0.05	0.82	27433	1800	76
7.5, 7.5, 15	74	11.31	0.04	0.63	28411	1800	75
7.5, 15, 25	70	13.95	0.04	0.56	39476	2500	70

MU: Monitor Units

TD: Prescribed Dose

**Table 8:** Postshielding peripheral dose in the region of the pubic symphysis, expressed as a percentage of the monitor units and as a percentage of the prescribed dose.

Collimator size (mm)	Distance (cm)	Postshielding peripheral dose (cGy)	Postshielding peripheral dose (%MU)	Postshielding peripheral dose (%TD)	Monitor units	Prescribed dose (cGy)	Isodose line (%)
12.5	63	1.24	0.04	0.06	3483	2200	80
25	71	3.94	0.04	0.20	10355	2000	70
20, 20, 10	72	5.75	0.04	0.36	12911	1600	70
12.5, 12.5, 10	70.5	4.62	0.03	0.36	13271	1300	70
20, 20, 7.5	71	5.66	0.04	0.40	14289	1400	70
15	67	5.34	0.04	0.36	14621	1500	70
35	83	6.66	0.04	0.37	15061	1800	70
12.5, 12.5, 7.5	82	7.26	0.04	0.40	20391	1800	70
10	87	6.23	0.03	0.35	20789	1800	70
5, 5, 5	68	4.64	0.02	0.23	20798	2000	70
7.5	82	8.06	0.04	0.37	20969	2200	70
10, 10, 5	74	9.18	0.04	0.44	24249	2100	70
5	76	7.26	0.03	0.40	24680	1800	80
10, 12.5, 20	70	10.04	0.04	0.63	26656	1600	75
5, 5, 10	74	11.76	0.04	0.65	29230	1800	70
5, 10, 12.5	73	11.52	0.04	0.61	29833	1900	70
5, 5, 5	80	11.64	0.04	0.55	32202	2100	70
7.5, 12.5, 20	69	13.08	0.04	0.65	33130	2000	70
5, 7.5, 12.5	73.5	18.66	0.04	1.04	42177	1800	70
7.5, 12.5, 12.5	88	13.89	0.03	0.51	43623	2700	75

MU: Monitor Units

TD: Prescribed Dose

The first two columns of each table list the collimator size used in each path of the treatment and the distance of the dosimeter from the treatment center. The next three columns list the PD in cGy, as a percentage of MU (% PD in cGy/MU) and as a percentage of the prescribed dose (% TD). Furthermore, the number of MU, and the TD to the isodose line (IL) delivered in each treatment, are listed.

The mean preshielding PD was 51.79 cGy (54.13 cGy), 13.31 cGy (7.61 cGy) and 10.07 cGy (5.77 cGy), in the thyroid gland, the umbilicus and the pubic symphysis, respectively, since in the preshielding measurements the nipple was not monitored. The mean postshielding PD was 38.40 cGy (33.13 cGy), 12.18 cGy (5.72 cGy), 10.94 cGy (4.83 cGy), and 8.69 cGy (3.90 cGy), in the thyroid gland, the nipple, the umbilicus and the pubic symphysis, respectively. The standard deviation of mean PD measurements is given in parenthesis. Cyberknife mean PD to extracranial sites ranged from 0.22% of the delivered number of MU, for the thyroid gland, to 0.04% for the pubic symphysis.

### Influence of the monitor units and collimator size on the peripheral dose

**Figure 2:**
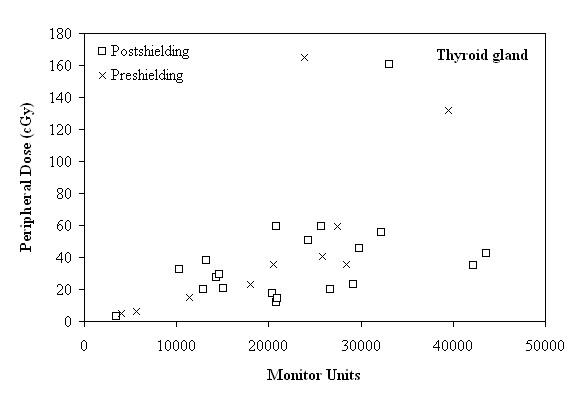
**Peripheral dose (cGy) as a function of the monitor units, in the region of the thyroid gland, before and after shielding**.

**Figure 3:**
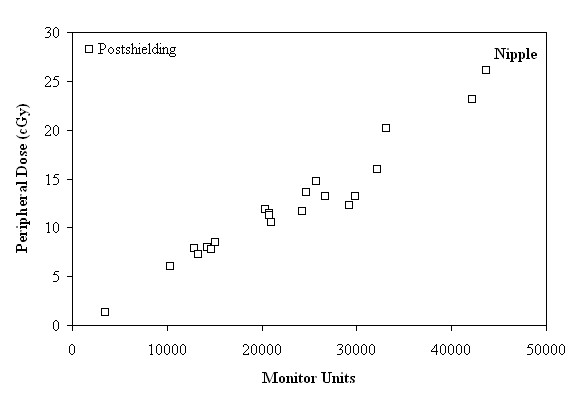
**Peripheral dose (cGy) as a function of the monitor units, in the region of nipple, after shielding**.

**Figure 4:**
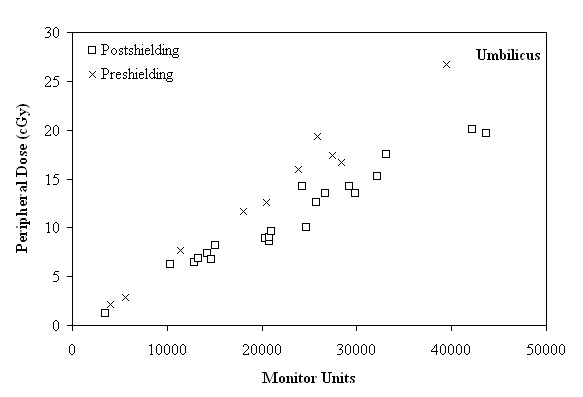
**Peripheral dose (cGy) as a function of the monitor units, in the region of umbilicus, before and after shielding**.

**Figure 5:**
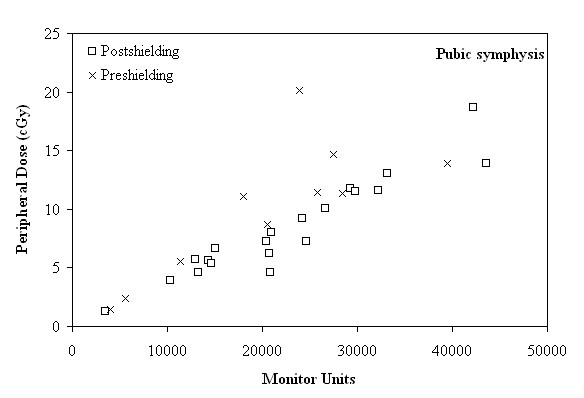
**Peripheral dose (cGy) as a function of the monitor units, in the region of pubic symphysis, before and after shielding**.

The PD measurements as a function of the MU in the four extracranial sites are presented in Figures 2, 3, 4 and 5. It is evident that the PD is proportional to the number of MU and this correlation can be utilized to estimate the PD, during intracranial treatment. In the thyroid gland (Figure 2) a correlation between PD and MU cannot be inferred, since the thyroid gland is located at different distances from the target in each patient (from 14 to 22 cm), and at such small distances from the target, even small changes in distances can lead to relatively large changes in PD.

As seen in Figures 3, 4 and 5, for distances larger than 29 cm, where the nipple, the umbilicus and the pubic symphysis are located, the major factor affecting the correlation between the MU and the PD is the distance of the anatomical region from the target. More specifically, the reduction of the PD (expressed as a percentage of MU) between the anatomical regions of the umbilicus and the pubic symphysis, can reach up to 22.54% and 23.39%, before and after shielding, respectively.

**Figure 6:**
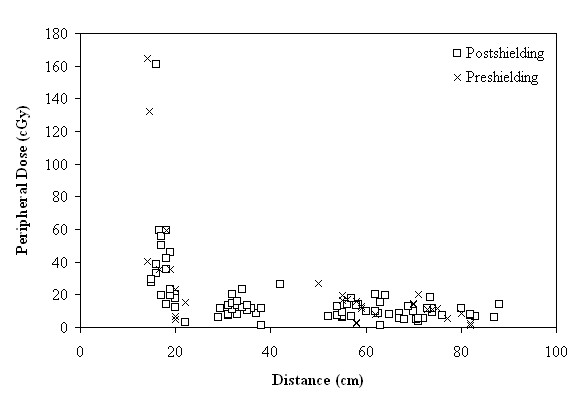
**Peripheral dose (cGy) as a function of distance for all measurements, before and after shielding**.

In Figure 6 the effect of distance on the PD is demonstrated in all the measurement data. It is evident that in the thyroid region PD is greatly affected by the distance while in the other regions it is almost constant.

**Figure 7:**
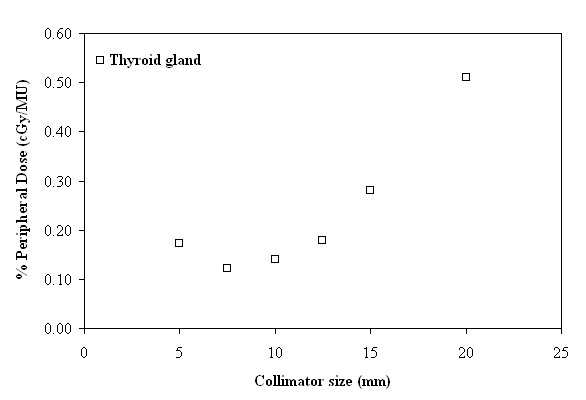
**Peripheral dose, expressed as a percentage of monitor units (%PD in cGy/MU), as a function of collimator size (mm), in the region of thyroid gland**.

**Figure 8:**
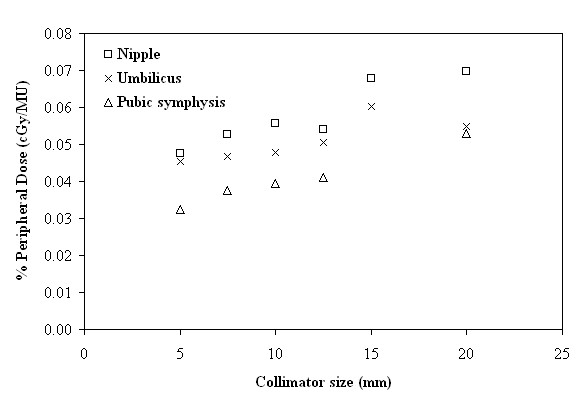
**Peripheral dose, expressed as a percentage of monitor units (%PD in cGy/MU), as a function of collimator size (mm), in the regions of the nipple, umbilicus and pubic symphysis**.

In order to examine the effect of the collimator size to the PD, the mean PD of all paths that used the same collimator size was calculated (after shielding). In Figures 7 and 8 it is evident that the increase of the collimator size corresponds to an increase of the PD. This increase is more apparent in regions near the tumor site, since PD is predominantly due to patient scatter radiation which is proportional to the scattering volume defined by the collimator size, and becomes less significant at larger distances, where PD is mainly attributed to head leakage and collimator scatter.

## Discussion

### Peripheral dose measurements

SRT procedures deliver a very high dose per fraction and as a result, the corresponding PD could be a limiting factor for long term surviving patients.

The mean preshielding PD in the thyroid gland site, as shown in Table 2 is 51.79 cGy and if expressed as a percentage of MU (or as a percentage of TD), is 0.22% (2.32%), while the corresponding mean postshielding result (Table 3) is 38.40 cGy, and if expressed as a percentage of MU (or as a percentage of TD) is 0.17% (2.06%). The fact that these results are higher compared to previous studies for both preshielding [11,13] and postshielding [12,13] measurements can be attributed to the fact that in these studies the thyroid gland has been considered as a structure of avoidance and beams passing though it were disallowed. In the present study, there was not such a constraint for the thyroid gland during the treatment planning procedure, thus the contribution of dose delivered by exiting beams increases the PD in this region.

The breast is considered to be one of the most sensitive organs to the carcinogenic effects of radiation, which can be affected by scattered radiation. The mean postshielding PD is 12.18 cGy according to Table 4, which is in good agreement with Zytkovicz et al. [10], and if expressed as a percentage of MU (or as a percentage of TD) it is 0.05% (0.65%), along a cranial caudal distance from 29 to 42 cm.

In the umbilicus site, PD could also have some potential deleterious effects on the fetus or embryo in the case of a pregnant woman being treated with radiotherapy [24,25]. Table 5 shows that the mean preshielding PD is 13.31 cGy and if expressed as a percentage of MU (or as a percentage of TD) is 0.06% (0.63%) along a cranial caudal distance from 50 to 62 cm. After shielding (Table 6), the mean PD is 10.94 cGy and if expressed as a percentage of MU (or as a percentage of TD) it is 0.05% (0.59%), respectively, along a cranial caudal distance ranging from 52 to 67 cm.

Finally, PD measurements in the pubic symphysis site are presented in Tables 7 and 8. The mean preshielding and the postshielding PD are 10.07 cGy and 8.69 cGy, respectively, for a cranial caudal distance of 67 to 88 cm. Mean PD expressed as a percentage of the number of MU (and as a percentage of TD), are 0.05% (0.48%) and 0.04% (0.47%), before and after shielding, respectively.

Previously published studies for the same anatomical regions report that the PD normalized to the delivered number of MU is 0.055% [11] to 0.074% [13], for 53 cm distance and 0.041% [11] and 0.049% [13] for distances of 71.0 and 75.5 cm, respectively.

It is evident that the shielding upgrade in the Cyberknife system creates a reduction in the PD in all extracranial sites of measurement. This is due to the fact that, with the placement of the shielding, an attenuation of leakage radiation and scattered radiation through the collimator is produced. The results showed that in the region of the thyroid gland the reduction in PD is 21.30% while Chaung et al [12] report a much greater reduction of 58.80%. Therefore, as mentioned above, the development of Cyberknife treatment plan without the thyroid gland to be identified as a structure of avoidance causes a reduction of the effect of shielding in this region.

More specifically, PD expressed as a percentage of the delivered MU, shows a reduction of 23.69% and 24.54%, in the umbilicus (a mean distance of 58 cm) and the pubic symphysis (mean distance of 75 cm), respectively. These results are in agreement with Chuang et al [12] who show a 25% reduction at 53 cm (lower thorax) and a 20% reduction at 71 cm (pelvis), while Di Betta et al. [13] report that the reduction of the PD due to the shielding upgrade could be up to 44% for the same anatomical regions.

### Influence of the monitor units and collimator size on the peripheral dose

In this study, the anticipated reduction of PD with an increasing distance from the treatment center is confirmed (Figure 6).

PD was expected to be proportional to the number of MU. However, the correlation between the number of MU and the PD can be utilized to roughly estimate the PD to a specific anatomical region of interest (for distances larger than 29 cm), during an intracranial treatment.

For distances larger than 29 cm, the PD expressed as a percentage of MU tends to be constant, with the values ranging as low as 0.02-0.08%, regardless of the existence of shielding.

After the installation of shielding, the delivered PD, in cases of treatments requiring 3000 to 15000 MU, ranged from 1.34 to 8.49 cGy and from 1.24 to 8.22 cGy, to the nipple and the umbilicus sites, respectively, according to Figures 3 and 4. If the treatment plan requires 20000 to 30000 MU, the delivered PD ranged from 10.54 to 14.79 cGy and from 8.59 to 14.25 cGy, respectively. Finally, the PD for treatment with more than 30000 MU up to 44000 MU ranged from 16.02 to 26.07 cGy to the nipple and from 15.24 to 20.07 cGy, to the umbilicus.

As can be seen in Figure 5, presenting the results of the pubic sypmhysis site, for a range of MU from 10000 to 15000, the delivered PD, ranged from 1.24 to 6.66 cGy. If the treatment plan requires 15000 to 30000 MU, the delivered PD ranged from 4.64 to 11.76 cGy. Finally, for a treatment plan which demands 30000 to 44000 MU, the corresponding values ranged from 11.64 to 18.66 cGy.

From Figures 7 and 8 it is evident that the increase of the collimator size corresponds to an increase of the PD, although a previous study [13] suggests that there is no significant difference. More specifically, for the 20 mm collimator with respect to the 5 mm one, the increase in PD can reach up to 196.88% close to the thyroid gland, while it is only 63.38% close to the pubic symphysis. On the other hand, as can be observed from Figure 7, the increase of PD in the smallest collimator could be attributed mainly to leakage radiation, which is governed by the number of delivered MU, but also to contributions from internally scattered radiation and scattered radiation from the collimators. A more extensive inquiry on the behaviour of the smallest size collimator may be needed.

### Estimation of the risk of induction of stochastic effects

According to the International Commission of Radiation Protection [26] the nominal risk coefficient (cases per 10000 persons per Sv) for radiation induced cancer is 33 and 112, for the thyroid and the breast, respectively. Making the assumption of considering the measured values of PD as the organ dose, this corresponds to a probability of secondary cancer appearance of about 0.127% for the thyroid and 0.136% for the breast. In the case of the thyroid gland, which is close to the treatment area, this probability could increase dramatically if some of the exit beams pass through it. The risk for inducing secondary cancers can be considered low for the organs studied, by taking into account the existing pathology of the patients undergoing Cyberknife treatment. However, it should not be completely disregarded, especially in long term surviving patients, who are being treated for benign diseases or for curatively non metastatic malignancies.

## Conclusions

Since stereotactic radiosurgery/radiotherapy procedures deliver a very high dose per fraction, the corresponding PD is a limiting factor for the long term surviving patients. Taking into consideration that more patients are now being cured of benign and malignant diseases, increased attention is required with respect to the late-onset of secondary cancers and damage to other organs. The PD measurements in this study, during intracranial treatment with Cyberknife, show that the possible risk of stochastic effect is low. However, a question that has yet to be answered is whether the thyroid gland should be a structure of avoidance in the treatment plan, especially if the treatment corresponds to a benign disease. Weighting the effect of the number of MU and the collimator size, can be effectively used during the optimization procedure, in order to choose the most suitable treatment plan that will deliver the maximum dose to the tumor, while being compatible with the dose constraints for the surrounding organs at risk.

## List of abbreviations

ΔV: Voltage Difference; CF: Calibration Factor; CR: Correction Factor; IL: Isodose Line; MOSFET: Metal Oxide Semiconductor Field Effect Transistor; MU: Monitor Units; PD: Peripheral Dose; SRS/SRT: Stereotactic Radiosurgery/Radiotherapy; TD: Prescribed dose.

## Competing interests

The authors declare that they have no competing interests.

## Authors' contributions

VV: contributions to conception and design, acquisition of data, analysis and interpretation of data; involvement in drafting and reviewing the manuscript. CA: contribution to acquisition and interpretation of data; involvement in reviewing the manuscript. HD: contributions to analysis and interpretation of data; involvement in drafting and reviewing the manuscript. AT: contribution to acquisition of data; involvement in reviewing the manuscript; NS: contribution to acquisition of data; involvement in reviewing the manuscript; DK: contributions to conception and design; involvement in reviewing the manuscript; GP: contributions to conception and design, interpretation of data; involvement in reviewing the manuscript.

All authors read and approved the final manuscript.
